# Stability in Aqueous Solution of a New Spray-Dried Hydrocolloid of High Andean Algae *Nostoc sphaericum*

**DOI:** 10.3390/polym16040537

**Published:** 2024-02-16

**Authors:** David Choque-Quispe, Carlos A. Ligarda-Samanez, Yudith Choque-Quispe, Sandro Froehner, Aydeé M. Solano-Reynoso, Elibet Moscoso-Moscoso, Yakov Felipe Carhuarupay-Molleda, Ronald Peréz-Salcedo

**Affiliations:** 1 Water and Food Treatment Materials Research Laboratory, Universidad Nacional José María Arguedas, Andahuaylas 03701, Peru; ychoque@unajma.edu.pe; 2 Department of Agroindustrial Engineering, Universidad Nacional José María Arguedas, Andahuaylas 03701, Peru; caligarda@unajma.edu.pe (C.A.L.-S.); rperez@unajma.edu.pe (R.P.-S.); 3 Research Group in the Development of Advanced Materials for Water and Food Treatment, Universidad Nacional José María Arguedas, Andahuaylas 03701, Peru; 4 Nutraceuticals and Biopolymers Research Group, Universidad Nacional José María Arguedas, Andahuaylas 03701, Peru; amsolano@unajma.edu.pe; 5 Food Nanotechnology Research Laboratory, Universidad Nacional José María Arguedas, Andahuaylas 03701, Peru; eibetmm22@gmail.com; 6 Department of Environmental Engineering, Universidad Nacional José María Arguedas, Andahuaylas 03701, Peru; 7 Department of Environmental Engineering, Federal University of Parana, Curitiba 80010, Brazil; froehner@ufpr.br; 8 Department of Basic Sciences, Universidad Nacional José María Arguedas, Andahuaylas 03701, Peru; ycarhuarupay@unajma.edu.pe

**Keywords:** stability, high Andean algae, hydrocolloid, SDH solution

## Abstract

There is a growing emphasis on seeking stabilizing agents with minimal transformation, prioritizing environmentally friendly alternatives, and actively contributing to the principles of the circular economy. This research aimed to assess the stability of a novel spray-dried hydrocolloid from high Andean algae when introduced into an aqueous solution. *Nostoc sphaericum* freshwater algae were subject to atomization, resulting in the production of spray-dried hydrocolloid (SDH). Subsequently, suspension solutions of SDH were meticulously prepared at varying pH levels and gelling temperatures. These solutions were then stored for 20 days to facilitate a comprehensive evaluation of their stability in suspension. The assessment involved a multifaceted approach, encompassing rheological analysis, scrutiny of turbidity, sedimentation assessment, ζ-potential, and measurement of particle size. The findings from these observations revealed that SDH exhibits a dilatant behavior when in solution, signifying an increase in with higher shear rate. Furthermore, it demonstrates commendable stability when stored under ambient conditions. SDH is emerging as a potential alternative stabilizer for use in aqueous solutions due to its easy extraction and application.

## 1. Introduction

Suspension stabilizers find widespread applications in various industrial processes, ranging from the formulation of consumable foods, and pharmaceuticals, to water treatment. Maintaining the suspension of solids in an aqueous medium not only enhances the visual quality of foods and drugs [1,2] but, in water treatment, it also improves the efficiency of removing both suspended and dissolved materials. Achieving these objectives relies on the utilization of stabilizing agents, which can be of chemical origin or derived from biological origin [3,4]. Nevertheless, the use or production of these stabilizers may generate waste, posing potential environmental challenges. Consequently, there is a growing interest in developing stabilizers from biological origins that are environmentally friendly and contribute to the principles of the circular economy.

A diverse array of stabilizing agents, including guar gum, xanthan gum, sodium alginate, pectins, carrageenans, gelatins, and locust bean gum, are extensively employed due to their exceptional functionality. These ingredients have significantly transformed the landscapes of the food and pharmaceutical and water treatment industries, with their availability in the market reflecting their synthetic, semi-synthetic, or natural origins [3,5,6,7]. With a heightened consumer awareness of environmental conservation and sustainability, there is a discernible preference for natural hydrocolloids, with algae emerging as prominent contenders in this category [8,9].

On the other hand, the extraction processes of commercial gums or hydrocolloids in many cases require the use of organic solvents and strong acidic or basic media [10,11], generating waste that negatively impacts the environment. Given this, the proposal of extraction with environmentally friendly methods is recurrent, so an alternative is extraction by atomization, and nostoc, being an algae with high moisture content (around 98%), is ideal to be subjected to this process.

While the majority of marine algae have undergone extensive study and exploitation in the food industry, the scientific knowledge and utilization of freshwater algae, particularly their extracted derivatives like the hydrocolloids of *Nostoc sphaericum*, remain limited. *Nostoc sphaericum*, an algae thriving as a renewable natural resource in the lagoons and wetlands of the Peruvian Andes, represents an area of untapped potential [12,13,14]; it has a protein content of 24.01%, fat 1.88%, ash 6.19%, fiber 8.84%, carbohydrates 57.32%, humidity 10.57%, and pH 6.91 in dehydrated samples on a wet basis [14].

Introducing novel stabilizers with broad applications poses a formidable challenge for the current industry, and the exploration of high Andean algae, such as *Nostoc spaericum*, holds promise in meeting these expectations. However, understanding the key parameters of the stabilizers or hydrocolloids, including ζ potential, particle size, and rheological behavior, is imperative [5,15,16,17]. Additionally, comprehending their behavior during storage is essential. The stability of stabilizing agents in an aqueous medium over time is critical during application. This aspect becomes particularly significant in preventing the undesired sedimentation of suspended solids, a concern in various liquid products such as nectars, juices, and dairy-derived concentrates, and especially in the context of suspended drugs. Conversely, in applications where agglomeration is desired, such as certain processes [18,19,20,21,22], sedimentation becomes a favorable outcome.

The stability of hydrocolloids in aqueous solution hinges on various factors, including hydrocolloid concentration, pH, temperature, and mixing speed. This stability can be characterized by understanding key parameters such as ζ potential, particle size, molecular weight, shear stress, strain rate, and activation energy [18,19,20,21,22]. While these control parameters are well-established for commercial hydrocolloids, determining them for a new hydrocolloid is essential to define its potential.

In the high-altitude lagoons of Andahuaylas, a province of the Peruvian Andes, algae of the genus *Nostoc* flourishes at elevations above 4000 m. Remarkably, these algae serve as a folkloric food source for the residents of the local communities.

Despite its content of hydrocolloids with favorable techno-functional properties [14,23], this algae currently lacks commercial significance, potentially serving as a valuable alternative to commonly used industry hydrocolloids. The research is directed towards evaluating the aqueous stability of a newly developed spray-dried hydrocolloid derived from high Andean algae, specifically *Nostoc sphaericum*.

## 2. Materials and Methods

### 2.1. Raw Material

The samples of atomized hydrocolloid (SDH) of *Nostoc sphaericum* were supplied by the Laboratory of Research in Advanced Materials for Water Treatment of the National University José María Arguedas, Peru. They were extracted by atomization according to the methodology proposed by Choque et al. [14], which consists of liquefying the algae with distilled water in a 1/1 ratio, then being sieved at 45 microns, then atomized at 100 °C inlet temperature, air speed of 600 L/s, and suction speed of 38 m^3^/h in a mini spray dryer model B-290, Buchi brand (Flawil, Switzerland).

### 2.2. Preparation of Hydrocolloid Suspensions

**Figure 1:**
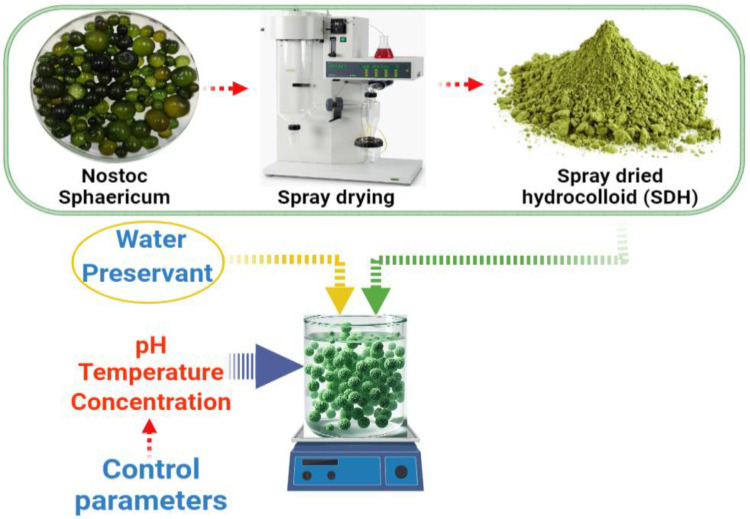
SDH solution preparation and analysis flowchart.

Suspensions were formulated in accordance with Table 1. The process involved adjusting the pH of distilled water with 0.1 M citric acid. Subsequently, 100 mL of the solution was taken, and 1 mg of SDH along with potassium sorbate 1 mg was added (added to prevent microbial growth). The mixture was stirred at 1500 rpm for 1 min for homogenization and then left in agitation at 60 rpm for 24 h. To achieve uniform temperature, the suspensions were heated at 60 and 80 °C under continuous agitation at 60 rpm until a constant temperature was reached (refer to Figure 1). Following this, the suspensions were cooled down to room temperature for subsequent evaluations.

### 2.3. SDH Characterization

The determination of the point of zero charge (PZC) involved preparing solutions with varying pH values (ranging from 2 to 12). In each case, 0.05 g of SDH was added to 50 mL of each solution and stirred at 150 rpm for 24 h at room temperature. The resulting solution’s pH was then measured, and the PZC was determined by identifying the intersection point of the initial pH and final pH curve [24].

In a separate analysis, SDH was subject to examination using a Thermo Fisher (Waltham, MA, USA) FTIR spectrometer in ATR mode. This analysis focused on identifying influential functional groups within the range of 4000 to 400 cm^−1^, with a resolution of 4 cm^−1^.

Additionally, X-ray diffraction analysis was conducted using a Bruker diffractometer, model D8-Focus (Karlsruhe, Germany), (Cu Kα1 = 1.5406 A°) at 40 kV and 40 mA, with a PSD Lynxeye detector. The degree of crystallinity was determined by calculating the ratio between the area corresponding to the crystalline phase and the total area under the XRD curve using Equation (1) [25].

(1) $$CD\;(\%)=\frac{S_{cr.p}}{S_{t}}\times 100,$$

Crystal size is not interchangeable with particle size, as crystals are contained within particles. Therefore, the average crystal size (*D*) was calculated from the diffractogram using Scherrer’s formula (Equation (2)) [26].

(2) $$D=\frac{k.\lambda }{\beta .cos\theta },$$

where k represents Scherrer’s constant (0.9), *λ* is the wavelength of the X-ray source (0.15406 nm), *β* denotes the peak width of the diffraction peak profile at half maximum height, a result of the small size of the crystallites (in radians), and *θ* signifies the position of the peak (in radians). The data were analyzed using Origin Pro 2023 software.

The morphology of the SDH was examined using a scanning electron microscope (SEM), particularly the Prism E model by Thermo Fisher (Waltham, MA, USA), operating at an acceleration voltage of 25 kV and a magnification of 1000×.

### 2.4. Analysis of Rheological Behavior

The experimental samples underwent continuous testing using an Anton Paar rotational rheometer, specifically the MCR702e model (Graz, Austria). The rheometer featured a concentric cylinder arrangement, and the tests were conducted at controlled shear rates ranging from 1 to 300 s^−1^ and at temperatures of 40, 60, and 80 °C. The acquired data were analyzed using shear stress models designed for non-Newtonian fluids, specifically the Power Law, Herschel–Bulkley, and Casson models, the details of which are presented in Table 2.

The rheological models underwent adjustment via non-linear regression, employing the least squares difference as the convergence criterion and evaluated using the Quasi-Newton (QN), Simplex/Quasi-Newton (SQN), and Rosenbrock/Quasi-Newton (RQN) methods [27,28]. To assess the model’s quality, key metrics including the adjusted correlation coefficient (R^2^), the residual mean square of the error (*MSE*) calculated using Equation (6), and the mean absolute percentage of the error (*MAPE*) determined through Equation (7) were considered.

(6) $$MSE=\frac{{\sum_{i}\left(x_{i}-{\hat{x}}_{i}\right)}^{2}}{n},$$

(7) $$MAPE=\frac{1}{N}\sum_{i=1}^{N}\frac{\left|x_{i}-{\hat{x}}_{i}\right|}{x_{i}}$$

In the given equations, $x_{i}$ represents the observed value; ${\hat{x}}_{i}$ denotes the predicted value; n signifies the number of observations, and N represents the totalnumber of experimental observations.

Similarly, an assessment of the dispersion of residuals was conducted, employing criteria such as Random (R), Slightly Random (SR), and Tendentious (T). Models demonstrating the best fit exhibited a random distribution of residuals.

These analyses were conducted at a significance level of 5%, utilizing Excel sheets, the Solver utility, and Statistica V12 software (Statsoft, Tulsa, OK, USA).

### 2.5. Determination of Temperature Dependence

The influence of temperature on the rheological behavior was examined by assessing the activation energy (*E_a_*), providing insights into the behavior of colloidal solutions, interpenetrating networks, and nanofluid flow. This parameter is linked to the energy necessary for the interchain displacement of polymers, with higher *E_a_* values indicating elevated crosslinking [29,30]. The calculation of *E_a_* was carried out using the Arrhenius equation (Equation (8)) based on the consistency index values.

(8) $$k=k_{0}e^{-\frac{E_{a}}{R}.\frac{1}{T}},$$

where *k* is the consistency index of the fitted model; *k*_0_ is the pre-exponential factor; *R* is the universal gas constant (8.314 kJ/kmol.K), and T is absolute temperature, K.

### 2.6. SDH Suspension Stability Evaluation

The stability of the suspensions was evaluated for 20 days, employing indicators such as turbidity, sedimentation, color, and ζ potential.

For each SDH suspension formulation, 20 mL was dispensed into flat-bottomed tubes with a diameter of 1.6 cm. Subsequently, 10 mL of suspension was extracted from the top of each treatment at rest, and turbidity was measured at intervals of 4 days for 20 days. The measurements were conducted by recording the transmittance at 560 nm using a UV-Vis spectrophotometer, specifically the Thermo Fisher Genesys 150 UV model (Waltham, MA, USA). Distilled water with potassium sorbate at the study pH served as a control. It is noteworthy that samples were discarded after each measurement.

To assess sedimentation, 8 mL of the tube’s remaining volume was extracted and discarded, leaving 2 mL. The residual content was vigorously vortexed at 3000 rpm for 2 min to achieve sediment homogenization. Subsequently, the homogenized sample was subject to spectrophotometric analysis, measuring transmittance at 560 nm. This process was repeated over a span of days, with readings taken at 4-day intervals.

The color stability of the suspensions was evaluated in the CIE L* a* b* color space, employing specific criteria. Luminosity (L*) was gauged on a scale from 0 = black and 100 = white, while chroma values a* and b* were utilized to determine color characteristics (+a = red, −a = green, +b = yellow and –b = blue) [31]. For this analysis, treatment samples were examined using a Konica Minolta colorimeter, model CR-5 (Japan), and readings were recorded in the reflectance module. Additionally, the color index (CI*) was calculated using Equation (9), providing a singular numerical representation of the color index as follows [32]:

- If CI* −40 to −20, colors range from blue-violet to deep green.
- If CI* −20 to −2, the colors range from deep green to yellowish green.
- If CI* −2 to +2, represents greenish yellow.
- If CI* +2 to +20, colors range from pale yellow to deep orange.
- If CI* +20 to +40, colors range from deep orange to deep red.

(9) $${CI}^{*}=\frac{a^{*}\cdot 1000}{L^{*}\cdot b^{*}},$$

For the ζ potential measurements, a 2 mL aliquot was extracted from each treatment and transferred to a polystyrene cell. Then, the samples were subject to analysis using dynamic light scattering equipment (DLS, Zetasizer ZSU3100, Malvern Instruments, Worcestershire, UK). The instrument operated at 632.8 nm, a scattering angle of 90°, and an electric field strength of 5 V/cm. To ensure accuracy and reproducibility, readings were performed in triplicate.

### 2.7. Statistical Analysis

The data on the stability properties of the SDH solutions were collected in Excel sheets and were evaluated by measuring the main effects and interactions of the input variables using the Statistica V12 software. The PCA analysis was carried out by standardizing the data of the response variables to integer values through the Origin Pro 2022b Software.

## 3. Results and Discussion

### 3.1. Zero Charge Point (ZCP)

**Figure 2:**
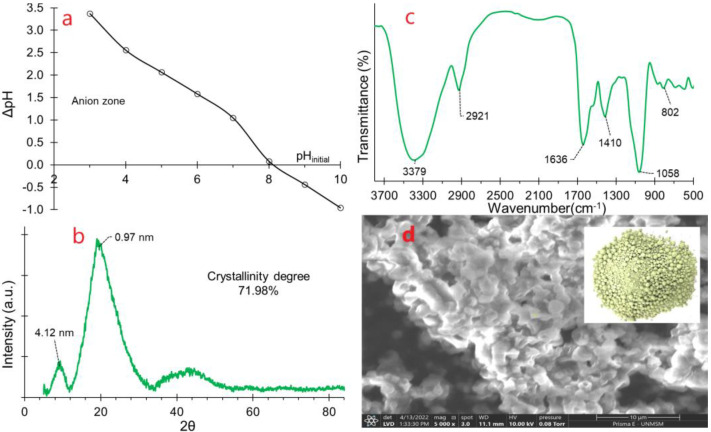
Characteristics of SDH (**a**) Zero charge point, (**b**) XRD diffractogram, (**c**) FTIR spectrogram, (**d**) SEM image.

The zero charge point (ZCP), also known as the isoelectric point, represents the pH value at which a substance in a solution carries no electrical charge. This parameter significantly influences the stability of substances in aqueous media [33,34]. Values below pH_ZCP_ indicate a higher availability of functional groups with a positive charge in the substance [33,35]. In the case of SDH, the reported pH_ZCP_ was approximately 8.1 (Figure 2a). This finding suggests that in the aqueous medium, SDH has the capacity to adsorb negatively charged molecules by physisorption or chemisorption at a pH lower than the pH_ZCP_.

### 3.2. Diffractometric Analysis and Degree of Crystallinity

Powdered materials are often favored for their enhanced stability and extended shelf life, especially when processing high crystallinity; this is in contrast to amorphous materials, which tend to retain more water [36,37], which could lead to microstructural collapse and microbiological instability [38,39].

Although the diffractogram of SDH reveals an overall amorphous structure (Figure 2b), two discernible peaks, at 9.17° and 19.52° of 2θ, respectively, indicate the presence of crystalline zones. These zones are associated with materials featuring non-covalent bonds and gelling qualities, such as CMC, citrus pectin, guar gum, and alginates among others [36,38,40,41,42,43].

The XRD pattern of SDH reports a degree of crystallinity at 71.98% and amorphous content at 28.02%, signifying a substantial level of crystallinity. This heightened crystallinity is likely attributed to the protein content within SDH [44], rendering it a stable material during storage and thereby enhancing its shelf life significantly [36,39].

### 3.3. FTIR Analysis

Fourier transform infrared analysis (FTIR) is instrumental in unraveling the intricate interactions involving stretching, bending, and torsion of chemical bonds within materials [14,45]. A pronounced peak of high intensity centered around 3400 cm^−1^. (Figure 2c) signifies the stretching of hydroxyl groups present in water, amides, carbohydrates, and carboxylic acids. Simultaneously, intense peaks at approximately 2900 cm^−1^ are indicative of vibrations from the methyl groups of carbohydrates, a characteristic feature of biological origin material [46].

The peak around 1600 cm^−1^ is attributed to the -OH stretching of adsorbed water molecules, suggesting a highly hygroscopic material as a result of its hydrocolloid content [7]. Furthermore, the peak at 1410 cm^−1^ is associated with the stretching of the C-O, C-H, and -OH single bonds, predominantly from carbohydrates [47].

Conversely, the peak observed at approximately 1050 cm^−1^ signifies the stretching of the C-O, C-O-C, and C-OH bonds within the polymeric chains of carbohydrates and proteins [2,47]. Peaks registering below 1000 cm^−1^ constitute the distinctive fingerprint of SDH, attributed to the stretching of the C-H and C-O bonds inherent to carbohydrates, a characteristic feature of hydrocolloids [47,48].

### 3.4. SEM Analysis

Particle morphology serves as a macroscopic representation of molecular arrangements within a material [49]. In the SEM image (Figure 2d), spherical shapes predominate, a characteristic tendency of materials containing carbohydrate-protein-lipid complexes. This configuration arises from interactions among nonpolar groups within the particle, leading to them agglomerating in spherical form [47]. The splashed or dented shapes in the photomicrograph are attributed to the rapid vaporization of water during the atomization process, causing contractions on the particle surface [43]. Such morphological features are traits of spray-dried materials [44,50].

### 3.5. Rheological Analysis

**Figure 3:**
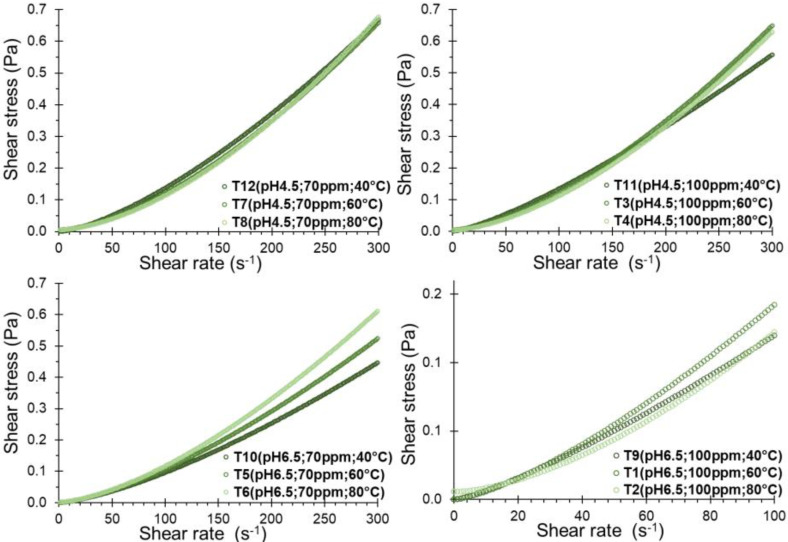
Rheological behavior of SDH in solution.

A noticeable increase in shear stress corresponding to shear rate was observed for all the formulations (Figure 3), displaying a consistent trend, especially among formulations with a pH of 4.5. Although the temperature allows the shear stress to be slightly increased, the opposite effect was observed at pH 6.5.

**Figure 4:**
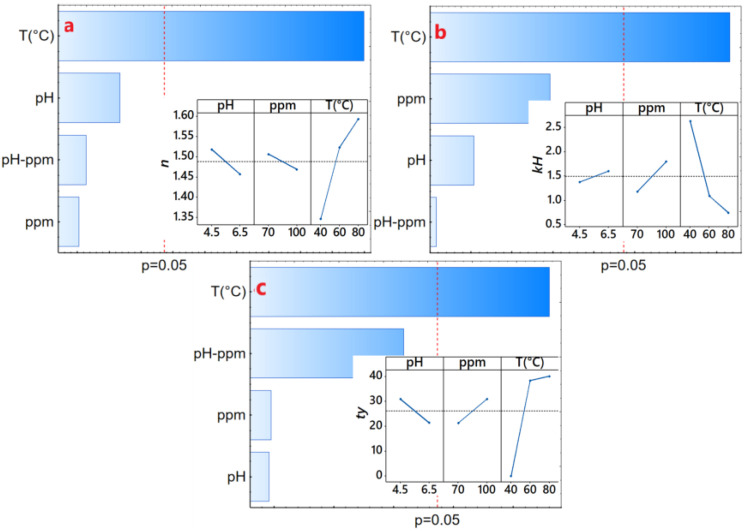
Effect diagram, (**a**) consistency index (k_H_), (**b**) behavior index (n), (**c**) elastic limit ($\tau_{y}$).

The concave curve fitted to the Power Law model yielded R^2^ values exceeding 0.9898, and the Herschel–Bulkley model resulted in R^2^ values surpassing 0.9929. In both models, behavior index values (n) exceed 1.0 (Table 3), which suggests that the solution behaves as a dilatant fluid. This behavior aligns with shear thickening dispersions [23,51,52], a characteristic observed when subject to a wide range of cutting rates [53,54,55]. Notably, this behavior intensified with significantly increasing temperature (Figure 4a), likely attributed to the thickening capacity of the hydrogels and gums [53,56,57], although no significant differences of n with SDH concentration and pH of the solution have been evidenced (*p*-value < 0.05).

The consistency index, denoted as *k* in the Power Law model or *k_H_* in the Herschel–Bulkley model, serves as an indirect measure of viscosity, indicating that as the fluid density increases, it becomes thicker or more viscous. Notably, a significant decrease in the consistency index was observed with increasing temperature (Figure 4b). It exhibited an increase with the concentration of SDH and pH of the solution (*p*-value < 0.05), implying that higher concentrations of SDH lead to thicker solutions, exemplified by higher values at concentrations of 100 ppm of SDH as seen in T1 and T9 (Table 3).

Regarding the elastic limit or yield point ($\tau_{y}$), no consistent trend was observed. However, in some instances, it increased with the temperature and the solute concentration (Figure 4c), indicating a greater shear stress requirement to initiate the deformation of the SDH solution. Higher values of $\tau_{y}$ have been observed for the Herschel–Bulkley model compared to the Casson model (Table 3). Though these values depend only on the fit of the model, both present the same trends.

**Figure 5:**
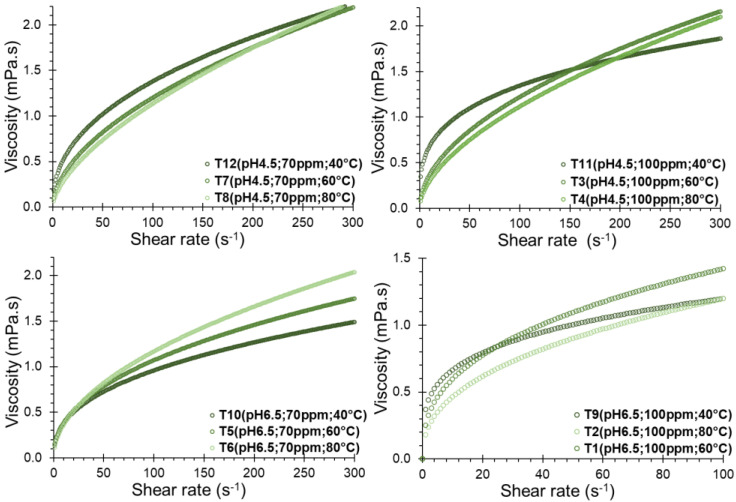
SDH viscosity in solution as a function of shear rate.

Concerning viscosity behavior, an increase was noted in correlation with the shear rate for all SDH formulations. However, no clear trend was evident with temperature (Figure 5). This indicates that as the shear rate increases, the fluid undergoes rapid deformation, exhibiting rheopectic behavior. This manifestation is indicative of crystallization induced by continuous shear, a characteristic feature of dilatant fluids [57,58]. As gelatinization progresses, SDH granules swell and become more deformable, contributing to this observed rheopectic behavior.

To study the changes of the hydrocolloid in solution, it is essential to heat the mixture under controlled conditions while continuously recording the viscosity changes over time [59]. This can be measured through the rapid viscoanalyzer (RVA); this method is characterized by a faster mixing action, which allows understanding of the rheopectic behavior through pasting curves, providing information on the initial gelatinization temperature and range, maximum or peak viscosity, swelling capacity (during heating), retrogradation and syneresis (during cooling), final viscosity or in a state of equilibrium, and capacity to form gels (in resistance to heat and mechanical agitation). These parameters allow us to know the useful life or the behavior of hydrocolloids in solution during storage [60,61].

**Figure 6:**
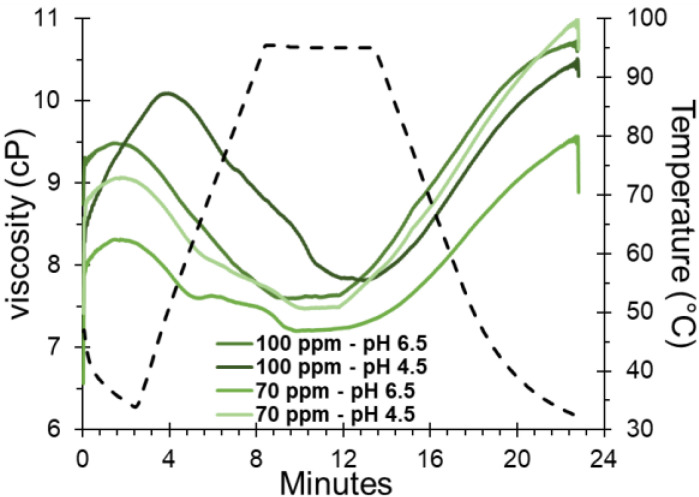
Rheopectic behavior of SDH in solution as a function of temperature.

The rheopectic behavior of SDH solutions becomes apparent with heating, as the apparent viscosity shows a tendency to increase over time. Notably, samples heated within the initial 4 min exhibit peak viscosity values, reaching levels between 8.5 to 10 cP in the temperature range of 60 to 90 °C (Figure 6). However, for durations exceeding 20 min, higher viscosity values are consistently reported across all aqueous formulations, particularly around 30 °C. This phenomenon can be attributed to the elevated content of total solids dissolved in the dispersant solution produces, leading to an increase in viscosity. This increase restricts intermolecular movement driven by hydrodynamic forces due to the complex and branched structure of SDH. Consequently, the solution establishes intermolecular bonds, contributing to the observed rise in viscosity [62].

### 3.6. Activation Energy of Solutions with SDH

The activation energy serves as a metric for quantifying the energy required to overcome resistance in the flow of a viscous fluid [30,63]. It can be calculated using the consistency index, an indirect measure of viscosity. Our study reveals a significant increase in *E_a_* in the presence of the SDH, which is more pronounced at higher pH levels. Specifically, at 100 ppm of SDH, the *E_a_* value was determined to be 37.74 kJ/mol (Table 4).

This rise in *E_a_* can be attributed to the increased viscosity of the solution. Consequently, a higher shear stress is required to induce deformation in the parallel plates of the water-SDH system. This observation underscores the interdependence of temperature and viscosity, a behavior commonly noted in various hydrocolloid materials and gums [64,65,66]. Elevated *E_a_* values signify a greater demand for external energy to facilitate molecular movement. This correlation aligns with heightened viscosity attributed to the presence of macromolecules with gel-forming capabilities, resulting in more stable during thermal processing and exhibiting non-Newtonian behavior [62,67,68,69]

### 3.7. SDH Suspension Stability

The stability of SDH was evaluated through the variation in turbidity and sedimentation over time. Undesirable occurrences such as phase separation, precipitation, and agglomeration of hydrocolloids in solution are due to the thermodynamic incompatibility between phases, and these are particularly problematic in industries like food where appearance is crucial [33,70].

These phenomena are influenced by various factors intrinsic to the hydrocolloid, including concentration, particle size, electric charge, solubility, molecular weight, and ionic strength. Simultaneously, the solvent medium is sensitive to pH and temperature conditions. Achieving stability necessitates a delicate balance of attractive and repulsive interactions between the hydrocolloid and the solvent medium [71,72].

**Figure 7:**
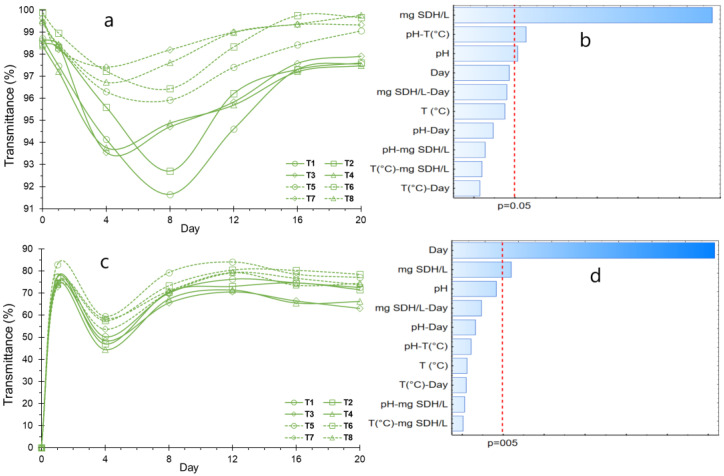
Suspension stability, (**a**) Turbidity variation; (**b**) Effects for turbidity; (**c**) Sedimentation variation; (**d**) Effects for sedimentation.

In Figure 7a, it is evident that there is high turbidity in the SDH solution on the initial day, signifying the extensive dispersion of SDH particles. Subsequently, from day 1 to day 8, there was a noticeable decline in transmittance, indicative of agglomeration, coalescence, and sedimentation of the hydrated SDH particles. From day 8 onwards, the transmittance increases, which translates into lower turbidity of the SDH solution. This suggests that the sedimented agglomerates are resuspended due to the hydration of the hydrocolloid molecules, producing a decrease in the sedimented particles, giving, as a result, high transmittance values for the sediments (Figure 7c). Notably, high transmittance values are reported for the sediments (Figure 7c). Furthermore, the observation from day 16 onwards indicates the stability of the SDH, suggesting that the hydrocolloid remains stable over extended periods. This stability is attributed to the hydration of SDH particles, establishing a delicate equilibrium between the attractive and repulsive forces governing the interactions between SDH particles and water.

The impact of the application dosage of SDH is notably significant in influencing the turbidity and sedimentation of the solution, as illustrated in Figure 7b,d. Higher concentrations exhibit reduced stability, attributed to the increased availability of SDH particles for agglomeration. It is worth noting that this susceptibility to agglomeration may also be influenced by the presence of additional constituents [71]. Conversely, pH emerges as a critical determinant of stability, with optimal stability observed in an acid medium, specifically at pH 4.5, Notably, significant differences among treatments are evident during the assessment of stability over various days, as detailed in Table 5.

### 3.8. ζ Potential and Particle Size of SDH Suspension

The stability of the SDH hydrocolloid particles in an aqueous medium was assessed through the ζ potential, a measure of the electrical charge indicating either repulsion or attraction [73]. Negative values ranging from −10 to −40 mV signify robust stability by fostering the repulsion of negatively charged particles. However, it is crucial to acknowledge that this stability is subject to influence from factors such as pH, concentration, and ionic strength [20,74,75].

**Figure 8:**
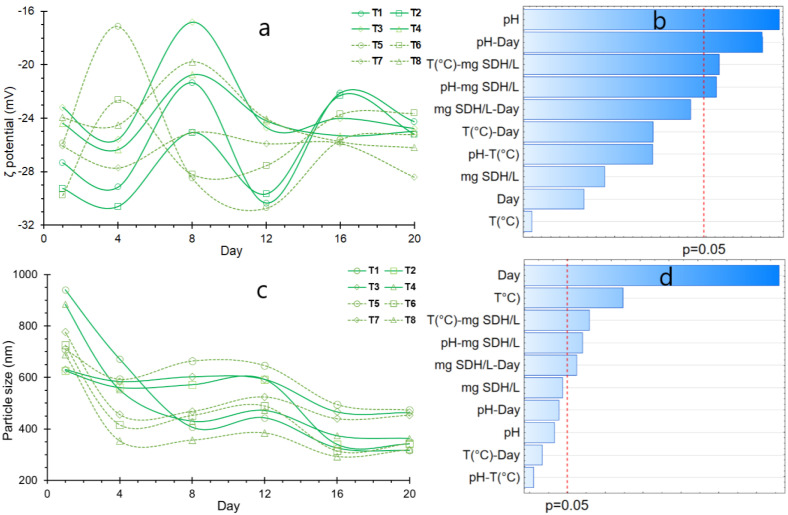
(**a**) variation of ζ potential; (**b**) Effects for ζ potential; (**c**) Variation of particle size; (**d**) Effects for particle size.

The SDH in solution displayed zeta potential values ranging between −17 to −31 mV, as depicted in Figure 8a. Initial values fell within the range of −23 to −30 mV, experiencing a notable increase by day 8. This upward shift suggests a decline in stability, as evidenced by the current rise in sedimentation (Figure 7a). After 20 days, zeta potential values were noted within the range of −24 to −28 mV, indicative of sustained stability for SDH throughout the storage period. Notably, there were significant differences between treatments, as indicated by the *p*-value (<0.05) in Table 6. These ζ potential align with observations in other hydrocolloids such as pectins, carrageenans, xanthan gum, and alginates, among others [14,20,76].

Its commendable stability is attributed to the abundance of negatively charged functional groups, such as carboxyl, carbonyl, and hydroxyl in SDH, as elucidated by the FTIR analysis (Figure 2c). The increase in pH enhances the prevalence of negative charges, augmenting the stability of SDH in solution, as evidenced by a significant effect (Figure 8b). Intriguingly, this contrasts with the behavior observed in gelation temperature and the impact of SDH addition, a departure from the typical response seen in analogous materials [74,77,78].

On the other hand, the particle size closely correlates with ζ potential, with nanometric sizes facilitating optimal dispersion of hydrocolloids and thereby promoting stability [21,22]. It has been observed that the particle size of SDH in solution decreases considerably with the progression of storage time (Figure 8c), initially ranging between 650 to 900 nm. By days 16 and 20, it fluctuates between 300 to 500 nm, displaying a tendency to maintain uniformity, albeit with slight differences observed between treatments (*p*-value < 0.05) (Table 6). This behavior signifies a high degree of dispersion for SDH, contrasting with relatively low values of ζ potential. Although no significant effect has been observed with pH, storage time and gelation temperature do have a considerable influence (Figure 8d).

### 3.9. PCA for the Treatments and Properties of the SDH Solution

**Figure 9:**
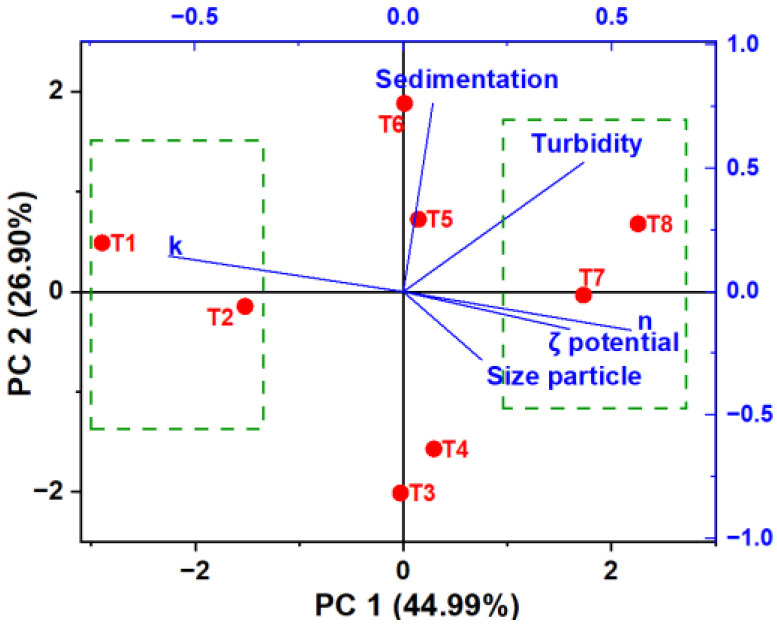
PCA for treatments.

PCA allows the exploration of data grouping based on principal components (PC) that explain the most significant variability in the dataset. Two primary components were identified, with the first PC1 (44.99%) grouping T1 and T2 on the left side of Figure 9. These treatments are notably influenced by the consistency index (k), indicating a tendency toward lower viscosity. On the right side, T7 and T8 are clustered, characterized by a pH of 4.5 and 0.07 g of SDH/L. These treatments exhibit higher turbidity, zeta potential, and particle size; these properties are desirable in aqueous food systems.

Additionally, PC2 (26.90%) reveals that T6 displays greater transmittance (measured as turbidity), while T4 and T3, situated in the negative part below, do not exhibit influential properties. This comprehensive analysis provides valuable insights into relationships between different treatments and their key properties.

Thus, the SDH hydrocolloid reported good stability in an aqueous medium, so this material could be used as an active ingredient in food products, pharmaceuticals, cosmetics, textiles, and paints due to the high solubility it presents. Likewise, it presents good stability against variations in pH (between 4.5 and 6.5) and temperature (between 60 and 80 °C). However, there are aspects to overcome, such as the residual color of SDH, which would considerably affect food products and drugs.

## 4. Conclusions

*Nostoc sphaericum*, a freshwater algae native to the Peruvian Andes, serves as a valuable source for extracting spray-drying hydrocolloid (SDH). This hydrocolloid exhibits favorable characteristics, rendering it highly stable in aqueous medium, with zero charge point at approximately pH 8.1, a crystallinity degree of 71.98%, and an average particle size of 4.12 nm. In the aqueous medium within the pH range of 4.5 to 6.5, SDH demonstrates dilatant behavior conforming to the Power Law (R^2^ > 0.99). Notably, its viscosity ranges from 8.5 to 10 cP at temperatures spanning 60 to 90 °C, displaying an activation energy fluctuating between 8.19 to 37.74 kJ/mol. Extended storage stability tests conducted up to day 20 reveal consistent turbidity, minimal sedimentation, ζ potential ranging between −31 to −17 mV, and particle size maintaining a steady range of 300 to 500 nm with low variability over time. Given these attributes, SDH emerges as a potential alternative stabilizer for application in aqueous media due to its exceptional stability. Although there is still the challenge of overcoming the depigmentation of the hydrocolloid, as well as the sensory study applied in food systems.

## Figures and Tables

**Table 1:** Experimental design matrix.

Treatment	Factor 1: pH	Factor 2: Concentration (ppm)	Factor 3: Temperature (°C)
T1	6.5	100	60
T2	6.5	100	80
T3	4.5	100	60
T4	4.5	100	80
T5	6.5	70	60
T6	6.5	70	80
T7	4.5	70	60
T8	4.5	70	80
T9	6.5	100	40
T10	6.5	70	40
T11	4.5	100	40
T12	4.5	70	40

**Table 2:** Rheological models for non-Newtonian fluids.

Model	Equation	Parameters	
Power law	$\tau =k\gamma^{n}$	k, n	(3)
Herschel–Bulkley	$\tau =\tau_{y}+k_{H}\gamma^{n}$	$\tau_{y},\;k_{H},\;n$	(4)
Casson	$\tau^{1/2}={\tau_{y}}^{1/2}+(k{\gamma )}^{1/2}$	$\tau_{y},\;k$	(5)

Donde: τ, yield stress (Pa); γ, shear rate, (s^−1^); k, consistency index (Pa.s^n^); n, behavioral index; $\tau_{y}$, elastic limit or yield point (Pa); $\eta_{B}$, plastic viscosity (Pa.s.); $k_{H}$, consistency index (Pa.s^n^).

**Table 3:** Parameters and statistical values of rheological models for SDH solutions.

Model	T1	T2	T3	T4	T5	T6	T7	T8	T9	T10	T11	T12
Power Law												
k(Pa.s^n^ × 10^−4^)	2.5042	1.7931	1.1154	0.7935	1.3742	1.1315	0.9657	0.6444	3.6937	1.5130	3.4568	1.8615
n	1.3772	1.4125	1.5196	1.5741	1.4457	1.5068	1.5473	1.6229	1.2557	1.4010	1.2951	1.4350
R^2^	0.9942	0.9898	0.9996	0.9999	0.9995	0.9997	0.9996	0.9999	0.9883	0.9998	0.9950	0.9986
MSE	0.0000	0.0000	0.0000	0.0000	0.0000	0.0000	0.0000	0.0000	0.0000	0.0000	0.0001	0.0001
MAPE	0.0892	0.1149	0.0703	0.0568	0.0495	0.0599	0.1522	0.0659	0.3849	0.0587	0.1107	0.0747
Residuals	R	R	R	R	R	R	R	R	R	R	R	R
EM	QN	QN	QN	QN	QN	QN	QN	QN	QN	QN	QN	QN
Herschel-Bulkley												
$\tau_{y}$(Pa × 10^−4^)	53.4769	56.4827	44.5463	31.8798	0.0000	18.7968	55.7519	53.5837	0.0000	0.0000	0.0000	0.1239
k_H_(Pa.s^n^ × 10^−4^)	1.2746	0.7209	0.9518	0.7023	1.3742	1.0556	0.7876	0.5272	3.6938	1.5131	3.4568	1.8579
n	1.5185	1.6041	1.5466	1.5949	1.4457	1.5187	1.5821	1.6573	1.2557	1.4010	1.2951	1.4354
R^2^	0.9961	0.9929	0.9997	0.9999	0.9995	0.9997	0.9997	0.9999	0.9968	0.9997	0.9958	0.9988
MSE	0.0000	0.0000	0.0000	0.0000	0.0000	0.0000	0.0000	0.0000	0.0000	0.0000	0.0001	0.0001
MAPE	0.0579	0.0990	0.0320	0.0216	0.0495	0.0452	0.4167	0.0179	0.3850	0.0587	0.1107	0.0745
Residuals	R	R	R	R	R	R	R	R	R	R	T	R
EM	QN	QN	QN	QN	QN	QN	QN	QN	QN	QN	QN	QN
Casson												
$\tau_{y}$(Pa × 10^−4^)	0.0109	0.0423	0.2735	0.4306	2.0557	0.3171	0.1104	0.0620	0.0158	0.3009	0.1378	0.0247
k(Pa.s^n^ × 10^−4^)	11.834	10.060	17.718	17.189	14.458	17.119	18.020	18.452	10.530	13.066	16.298	19.236
R^2^	0.9456	0.9426	0.9353	0.9270	0.9433	0.9385	0.9329	0.9211	0.9630	0.9545	0.9656	0.9507
MSE	0.0001	0.0001	0.0024	0.0026	0.0014	0.0021	0.0026	0.0032	0.0001	0.0008	0.0010	0.0020
MAPE	0.2130	0.2187	0.4176	0.5376	0.4910	0.4587	1.1148	0.5534	0.6942	0.4808	0.3843	0.4534
Residuals	T	T	T	T	T	T	T	T	R	T	T	T
EM	SQN	SQN	QN	SQN	SQN	SQN	QN	SQN	SQN	SQN	SQN	SQN

Where EM, estimation method; R, random; T, trending; QN, Quasi-Newton method; SQN, Simplex and Quasi-Newton method; MSE, mean square of the error; MAPE, mean absolute percentage of the error.

**Table 4:** Activation energy in SDH solutions.

Treatment			*E_a_* (kJ/mol)	R^2^
pH	ppm	T (°C)		
4.5	70	40	29.127	0.9708
4.5	70	60		
4.5	70	80		
4.5	100	40	37.043	0.9079
4.5	100	60		
4.5	100	80		
6.5	70	40	8.190	0.9144
6.5	70	60		
6.5	70	80		
6.5	100	40	37.740	0.981
6.5	100	60		
6.5	100	80		

**Table 5:** Significant difference between treatments for turbidity and sedimentation.

Treatment	Turbidity								Sedimentation							
	Day							*p*-Value *	Day							*p*-Value *
	0	1	4	8	12	16	20		0	1	4	8	12	16	20	
T1	a	a	c	e	d	d	c	<0.05	--	b	a	c	c	d	a	<0.05
T2	b	ab	b	e	c	d	c	<0.05	--	b	b	c	d	d	b	<0.05
T3	c	ab	c	d	c	cd	c	<0.05	--	c	c	e	e	e	c	<0.05
T4	d	a	c	d	c	d	c	<0.05	--	d	d	d	e	e	d	<0.05
T5	e	ab	ab	cd	b	bc	b	<0.05	--	a	e	a	a	b	e	<0.05
T6	f	b	a	bc	a	a	a	<0.05	--	c,d	f	b	b	a	f	<0.05
T7	g	ab	a	a	a	ab	ab	<0.05	--	e	g	c	b	c	g	<0.05
T8	h	ab	ab	ab	a	ab	a	<0.05	--	c,d	h	c	b	d	h	<0.05

* Different letters indicate a significant difference, evaluated through the Tukey test at 5%, n = 5.

**Table 6:** Significant difference between treatments for ζ potential and particle size.

Treatment	ζ Potential (mV)							Size Particle (nm)						
	Day						*p*-Value *	Day						*p*-Value *
	1	4	8	12	16	20		1	4	8	12	16	20	
T1	a	a,b	c	a	b	b	<0.05	a	a	c,d	c,d	c	b	<0.05
T2	a	a	b	a,b	b	b	<0.05	d	b	b	a,b	c	b	<0.05
T3	a	a,b,c	d	c	a,b	b	<0.05	d	a,b	a,b	a,b	a	a	<0.05
T4	a	a,b,c	c	c	a,b	b	<0.05	a,b	b,c	c,d	c,d	b,c	b	<0.05
T5	a	d	a	a	a	b	<0.05	c,d	a,b	a	a	a	a	<0.05
T6	a	c	a	a,b,c	a,b	b	<0.05	c,d	d,e	c	c	c	b	<0.05
T7	a	a,b,c	b	b,c	a	a	<0.05	b,c	c,d	c	b,c	a,b	a	<0.05
T8	a	b,c	c,d	c	a	a,b	<0.05	c,d	e	d	d	c	b	<0.05

* Different letters indicate a significant difference, evaluated through the Tukey test at 5%, n = 3.

## Data Availability

The data presented in this study are available in this same article.
